# Editorial: *In silico* gating mechanism studies and modulator discovery for MscL

**DOI:** 10.3389/fchem.2024.1376617

**Published:** 2024-02-09

**Authors:** Junmei Wang, Paul Blount, Tingjun Hou, Masahiro Sokabe

**Affiliations:** ^1^ Department of Pharmaceutical Sciences and Computational Chemical Genomics Screening Center, School of Pharmacy, University of Pittsburgh, Pittsburgh, PA, United States; ^2^ Department of Physiology, UT Southwestern Medical Center, Dallas, TX, United States; ^3^ Innovation Institute for Artificial Intelligence in Medicine of Zhejiang University, College of Pharmaceutical Sciences, Zhejiang University, Hangzhou, Zhejiang, China; ^4^ State Key Lab of CAD and CG, Zhejiang University, Hangzhou, Zhejiang, China; ^5^ Mechanobiology Laboratory, Nagoya University Graduate School of Medicine, Tsurumai, Nagoya, Japan; ^6^ Human Information Systems Laboratory, Kanazawa Institute of Technology, Hakusan, Ishikawa, Japan

**Keywords:** MscL, gating mechanism, lipid-MscL interaction, modulator design, molecular dynamics simulations

Mechanosensitive channel (MscL) proteins are viable pharmaceutical drug targets for development of precursors or antibiotics [Lane and Pliotas, Frontiers in Chemistry, 2023, 11; (Wang and Blount, 2023), Current Opinion in Physiology, 2023, 31] To this end, understanding the gating mechanism of MscL is necessary to achieve rational drug design. Although many research articles have been published in the last 10 years using variable techniques including FRET and the state-of-the-art molecular simulations techniques, there are still much unknown about the gating mechanism of this promising drug targets, for example, the fully open-channel MscL structure has not been experimentally determined and native ligands that modulate MscL are unknown or do not exist. Solving those mysteries can facilitate rational drug design modulators targeting MscL. The Research Topic focuses on elucidation of MscL gating mechanism using the start-of-the-art molecular dynamics simulations (MDS) which can be considered as a special microscope which can provide atomic details on the gating process. Of course, the findings by MDS need further validation using *in vitro/in vivo* experiments.

**FIGURE 1:**
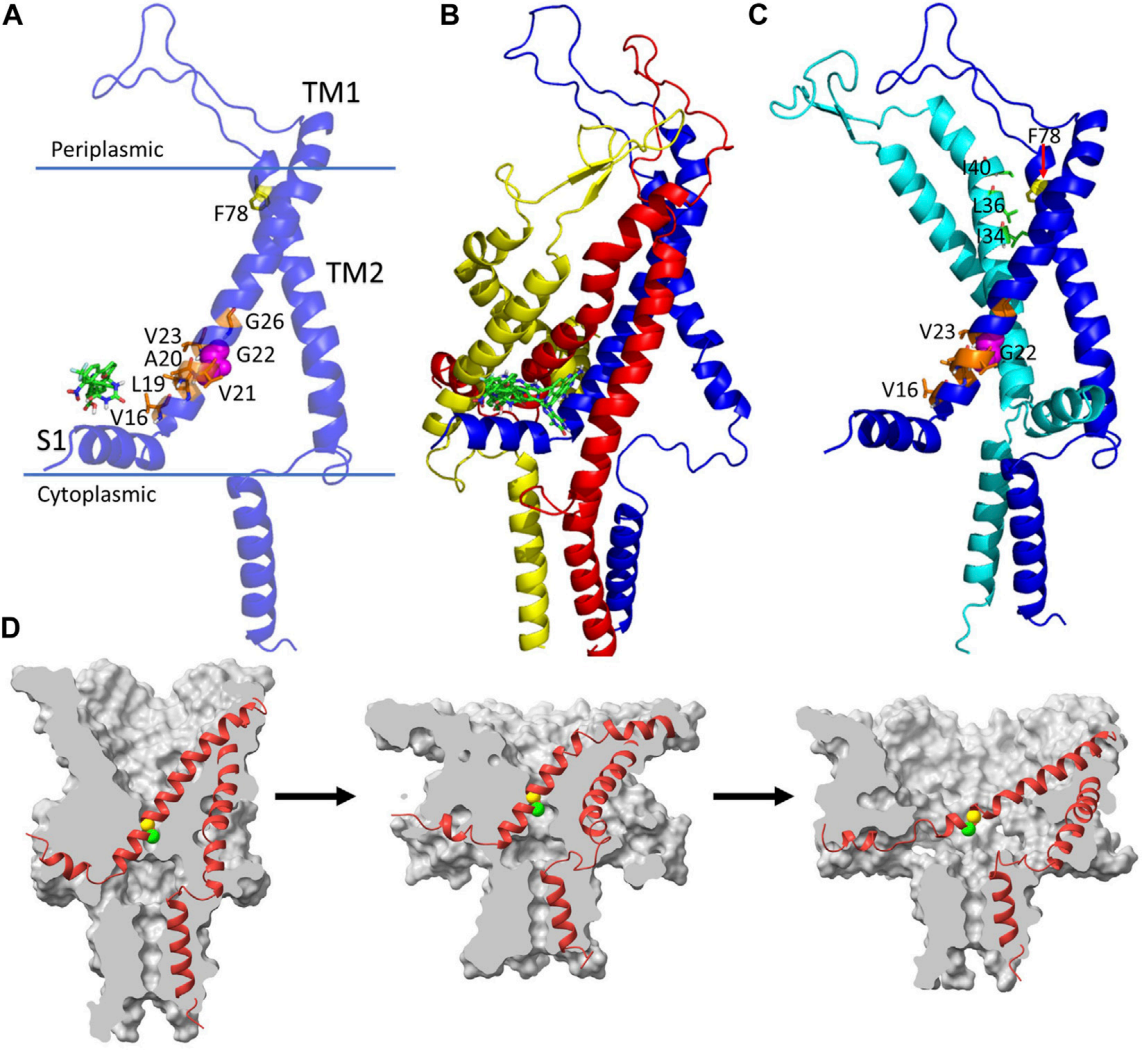
Structural basis for Mscl-based drug design and molecular mechanism of MscL channel gating. **(A)** a single unit of Ec-MscL with the hydrophobic residues at the pore constriction site shown as brownish sticks; G22, a key GOF residue and its G22N mutant displays spontaneous channel opening is shown in magenta sphere; F78 at TM2 (yellow sticks) and the N-terminal S1 domain correspondingly interact with lipids at the periplasmic and cytoplasmic sides and play crucial role in MscL opening. The binding site of drug molecules is shown using 011A and K05 (greenish sticks). **(B)** Five small molecules (011A, K05, 262, IRS-16 and SCH-79797) shown as green sticks are resided in a binding site formed by three Ec-MscL units. **(C)** Interaction between F78 and three hydrophobic residues (I34, L36 and L40) in TM1 of a neighbouring unit. The interaction mechanism was elucidated by Sawada et al. **(D)** Gating process revealed through large-scale MD simulations by Sharma et al. The channel undergoes an asymmetric silent expansion and TM1 bends [middle image of **(D)**] during the gating process. **(D)** was directly adopted from Sharma et al.’s paper (Figure 9).

The biological unit of a MscL is a pentamer which forms a helical bundle utilizing two transmembrane helices, TM1 and TM2 in each monomer (Figure 1A). From the periplasmic side, a larger loop connects TM1 and TM2, while in the cytoplasmic side, a bundle of five helices is formed (Sukharev et al., 2001). The N-terminal S1 domain as well as some TM2 residues (such as F78 of Ec-MscL) exposed to lipids can “feel” the lateral tension from membrane and initiate gating process (Iscla et al., 2008; Bavi et al., 2016). Other stimuli include small molecular modulators, ultrasound, pH, and temperature (Figure 1B). It is very well established that some hydrophobic residues including V16, L19, A20, V21, G22 and V23 form a constriction site which can stabilize the closed state of MscL (Figure 1A). The hydrophobic interactions are broken by a particular degree of membrane tension before the gate opens, thus, Blount and Moe described the hydrophobic pocket as a hydrophobic lock (Blount and Moe, 1999). Wetting the hydrophobic lock is considered as a rate-limiting step in the gating procedure (Jeon and Voth, 2008). Mutations occurring at those sites typically produce gain-of-function (GOF) MscL which are prone to gate-opening (Anishkin et al., 2005). G22N is a famous GOF mutant which displays spontaneously gate open in liposomes (Yoshimura et al., 2008).

In this Research Topic, Lane and Pliotas first briefly reviewed potential of MscL as a viable drug target including the structural basis of conducting rational drug design. Then they mainly focused on the triggers of MscL channel opening from three aspects, the modification of MscL protein itself via site-directed mutagenesis and post-translational modifications, the small molecular modulators and antimicrobials targeting MscL, and the alteration of membrane properties and components. All those stimuli can modulate the gating process, promising drug candidates which stabilize closed, expended or open states can be developed. The authors concluded that “Overall, there is great potential for new pioneering discoveries through the modulation of bacterial mechanosensitive channels in order to develop understanding of their structures, mechanisms, and functions but also for their use within biotechnology and as targets for antimicrobial therapies.”

Due to the lack of appropriate experimental means to obtain the atomistic open-channel structure of MscL, MDS becomes an indispensable tool to study the gating mechanism. Sharma et al. conducted long-time MDS for *Mycobacterium tuberculosis* MscL (TbMscL) for the wildtype and five GOF mutants (A20N, V21A, V21N, V21T and V21D). They performed multi-microsecond MD simulations with the TbMscL protein embedded in POPE membrane. The membrane tension was considered in two scenarios: only far-field tension applied to the entire simulation box was considered, and additional focusing forces were applied on lipids immediately surrounding the protein with the locally distributed tension protocol. The latter was applied to the wild type TbMscL as the channel pore did not open after 20 µs under high membrane tension with the first protocol. The authors found that the channel opening event is tightly associated with the disruption of the hydrophobic lock at the constriction region. Moreover, they proposed that funnel-shaped silent intermediate structures (Figure 1D, middle image) persist for one to serval or even tens microseconds prior to entering the fully open state. The intermediate structures were well characterized by the authors: the in plane-projected protein area is between 20 and 40 nm^2^, which is larger than the closed but smaller than the open structures; the constricted hydrophobic region remains dry; and TM1 bends more than 50° for the mutants and 65° for the wild type. Their MDS results not only are consistent with the previous computational and experimental studies, but also provide novel insights on the gating process.

Sawada et al. performed a combination of MDS and *in vitro* electrophysiology experiments for studying the gating mechanism of wild type Ec-MscL and three mutants, G22N, F78N and G22N/F78N. Instead of using far-field or locally distributed tension, they applied a negative pressure in the lateral axis of the membrane to simulate the membrane tension. It is known that G22N can cause gate spontaneously open in the absence of membrane stretch, while F78N mutation should not interfere with the spontaneous channel openings as F78 is considered as a membrane tension sensor. The authors hypothesized that the double mutation, G22N/F78N, would not affect the spontaneous channel openings. However, their patch clamp experiment results showed that both the stretch-dependent activation and the spontaneous channel openings were completely abolished in the double mutant. To explain the experimental results, they performed MDS and analyzed the force transduction pathway. Their simulation results nicely solve the mystery: the F78N mutation decreased its interaction not only with lipids, but also with a group of hydrophobic residues (I32, L36 and I40) in the neighboring TM1 helix (Figure 1C), leading to not only interference with the spontaneous gate openings via a slight tilting of TM1 toward the membrane plain, but also inefficient force transmission to the gate-constituting amino acids on TM1. This is the first demonstration of trans-subunit mechano-transduction critical for MscL gating opening, which may also facilitate cooperatively synchronized opening of subunits. Thus, the combination of MDS and *in vitro* experiment nicely enhance our understanding of the MscL gating mechanism.

The work by Wen et al. greatly expands the application of MscL in therapeutics. The authors expressed wild type Ec-MscL and two mutants (I92G/I19G and G26C) in different subcellular organelles in non-small cell lung cancer A549 cells. Then the authors conducted a series of *in vitro* and *in vivo* studies to investigate the potential application of activated MscL as a nanomedicine. They found that both the wild type and mutants can cause abnormal morphologies when they were expressed in mitochondrial inner membrane (MIM) of A549 cells, due to the increased permeability of the MIM; the MscL channel expressed in A549 cells can be activated by low intensity focused ultrasound (LIFU); and A549 tumors were suppressed by low sound pressure LIFU treatment. The authors also found that the I92G/I19G mutant is a better candidate as the mechanical transducer due to its adequate mechanosensitivity (i.e., larger than the wild type, but smaller than V23A) and more responsive to LIFU. Thus, the findings from this work provided insights into the mechanisms underlying non-apoptotic cell death and validated the possibility of applying nanochannel-based non-invasive ultrasonic strategy for cancer therapy.

In summary, the four papers collected in this Research Topic have significantly advanced our understanding of the MscL gating mechanism, furthermore, both papers running MDS to study gating mechanism utilized the number of water molecules in the gate region to quantitively measure gate open event. This novel approach can be applied to identify “true” modulators of MscL via MDS. The work by Wen et al. is a serendipity to this Research Topic. Their research findings validated a novel strategy for cancer therapy via ultra-sound activated MscL. Lane and Pliotas’s minireview nicely highlighted the current advances on MscL from many aspects, from gating mechanisms through mutation and post-translational modifications, modulation of MscL via membrane properties and components, to drug discovery targeting MscL. We expect those findings can facilitate the discovery of novel chemicals as a new generation of antibiotics, drug adjuvants and stimulus which can trigger the gating process for cancer therapy.
